# The neurobiological rationale for improving paternity leave in academia and beyond

**DOI:** 10.1177/23982128251401757

**Published:** 2025-12-12

**Authors:** Paul G Anastasiades, Valentina Mosienko, Antonia Tzemanaki

**Affiliations:** 1 School of Psychology and Neuroscience, University of Bristol, Bristol, UK; 2 School of Engineering Mathematics and Technology, University of Bristol, Bristol, UK

**Keywords:** Public engagement, British neuroscience association, neuroscience, parental leave

**Figure 1.**
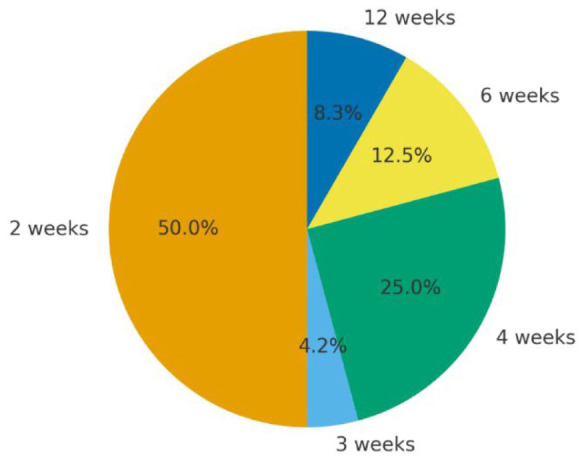
Distribution of paternity/partner leave duration across the UK Russell Group. Amount of paternity/partner leave allowance (in weeks) provided by the 24 UK higher education institutions that comprise the Russell Group based on publicly available information at the time of publication.

There is currently a growing public debate on paternity leave in the UK. This discussion has been driven by the evolving nature of modern parenthood, with fathers now taking on a far more active and engaged role in childcare than their predecessors (Dotti Sani and Treas, 2016; Gauthier et al., 2004). Despite these societal shifts, the UK continues to offer some of the least generous paternity or partner leave entitlements in Europe, a situation that risks perpetuating gender roles rather than supporting genuine gender equality. Recent government analysis has revealed that shared parental leave, designed to offer new parents more time with their family, has been ineffectual (Shared Parental Leave (SPL) evaluation, 2023). While some institutions, including in academia, have taken it upon themselves to enhance paternity leave offerings, most still receive the statutory 2 weeks (Figure 1). There are many reasons to think the current status quo is unacceptable, but what does the brain tell us? Much like attitudes to childcare, there is a growing consensus that male brains undergo pronounced alterations in response to their offspring. These changes coincide with hormonal shifts (Gordon et al., 2010; Storey et al., 2000) and are highly experience-dependent (Feldman et al., 2019) – for example, in same-sex male couples who had babies by surrogacy, the partner that takes on the greater childcaring role exhibits brain changes typically only observed in mothers (Abraham et al., 2014). Importantly, these changes are independent of whether the father is the child’s biological parent and correlate with time spent caring for their newborn. Changes in the paternal brain occur within a ‘caregiving brain network’ that supports empathy, social communication, and mentalising the needs and beliefs of others, allowing new parents to better interpret and plan for the ever-changing requirements of their children (Feldman et al., 2019). These neural adaptations are absent in non-parents, and child-evoked changes in neural activity are specific to a parent’s own infant (Feldman et al., 2019; Kuo et al., 2012; Nishitani et al., 2017). Alterations to the paternal brain are also lifelong, with brain scans of neurotypical septuagenarians able to differentiate between parents and non-parents (Orchard et al., 2020). Paternity-induced adaptations may also be neuroprotective, with studies showing reduced brain aging and improved memory in both animal models and UK Biobank data (Gatewood et al., 2005; Kinsley et al., 1999; Ning et al., 2020).

Giving fathers more time off in the immediate postpartum period also offers huge mental health benefits for the whole family. New parents are at increased risk of developing adverse mental health during the early postnatal period, with more than 40% new fathers reporting increased stress and anxiety symptoms (Philpott et al., 2022), and 10%–20% experiencing paternal postnatal depression (Philpott and Corcoran, 2018; Scarff, 2019). Postnatal depression also affects about 10% of new mothers, with 70%–80% experiencing transient ‘baby blues’ (Manso-Córdoba et al., 2020). Although the risk factors for postnatal depression are numerous and complex, partner support during the early postnatal period substantially reduces both the risk and severity of postnatal depression (Dennis and Ross, 2006; Misri et al., 2000; Pilkington et al., 2016; Ruan and Wu, 2024). This protective effect likely permeates through the family unit given the partners of women with postnatal depression are themselves more likely to suffer (Wain et al., 2025) and the children of mothers living with postnatal depression potentially developing lifelong cognitive and behavioural issues (Brookman et al., 2023; Rigato et al., 2022; Tambelli et al., 2015).

Analysis into the impact of perinatal mental health in the UK reveals an annual lifetime cohort cost of £8.1 billion (Bauer et al., 2022), one-fifth of which is born by the taxpayer via the already overstretched NHS and social services. Importantly, this analysis does not include the impact on paternal mental health, which is often underreported, likely due to a primary focus from both a public health and research perspective on the mother and child (Short et al., 2023) and general poor engagement with mental health services among men (Roxo et al., 2021; Seidler et al., 2016; Twomey et al., 2015). Symptoms of postnatal depression are often insidious, creeping up on unexpectant fathers over a protracted period within the first 6 months to a year post birth (Scarff, 2019). Sleep is a known risk factor for postnatal depression (Gallaher et al., 2018), and many babies do not begin to adopt normal sleep patterns until 3–6 months of age (Galland et al., 2012; Sadeh et al., 2009). Although we cannot do much about how well a parent’s child sleeps at night, even if we ignore how sleep deprivation will influence performance at work via effects on cognitive functions such as emotional regulation, attention, and executive control (Krause et al., 2017), is forcing new parents to wake early for a long commute the next day really best for their mental health?

Partner support during and after pregnancy may be particularly relevant in academia and related careers where there is a strong emphasis on moving away from traditional sources of family assistance to access a limited pool of job opportunities. Many academics are therefore ‘on their own’ in the immediate postpartum period, and so the impact of increased partner availability may be more keenly felt. Providing dedicated, paid partner leave facilitates greater input during the early weeks and months, strengthening the paternal bond via the neural adaptations described earlier, with lifelong consequences for paternal involvement in childcare (Norman et al., 2024). A corollary of this is the subsequent freeing of mothers from that burden of responsibility (Di Leo et al., 2025), helping retain more women in academia and stopping the ‘leaky pipeline’ that sees so many talented individuals choose to leave (Heidt, 2025; Powell, 2021). Based on this evidence, it is imperative that society act now to better support new families. Such changes have the potential to benefit both parents and children, creating a greater sense of belonging for new parents, leaving behind traditional gender roles that negatively impact the careers of new mothers, and improving efficiency at the workplace while reducing burdens on health services by improving mental health outcomes. Providing improved paternity/partner leave may therefore prove to be a powerful and relatively low-cost tool to build a more equitable society, within and beyond academia.
